# *Candida auris* Invasive Infection after Kidney Transplantation

**DOI:** 10.1155/2022/6007607

**Published:** 2022-01-28

**Authors:** Javier Reque, Rosa Arlandis, Nayara Panizo, Maria José Pascual, Alejandro Perez-Alba

**Affiliations:** ^1^Nephrology Department, Hospital General Universitario de Castellón, Castellón de la Plana, Spain; ^2^Nephrology Department, Hospital Clínico Universitario Valencia, Valencia, Spain

## Abstract

**Introduction:**

*C. auris* has been associated not only with a variety of invasive fungal infections, including candidemia, sometimes related to central venous catheter, but also with pericarditis and respiratory tract and urinary tract infections.

**Materials and Methods:**

We describe the case of a patient with persistent fever despite antibiotics, who presented with *Candida* isolation in blood cultures, typified as *Candida auris* species.

**Results:**

A 57-year-old male receiving peritoneal dialysis underwent kidney transplantation which was complicated by primary nonfunction due to arterial thrombosis necessitating graft nephrectomy. During the postoperative period, he presented with *Pseudomonas aeruginosa* pneumonia that was treated with levofloxacin and catheter-related *Enterococcus faecalis* bacteremia treated with linezolid. After hospital discharge, he then presented with herpes zoster infection treated with valacyclovir. Ten days later, he developed peritonitis and exit site infection with multidrug-resistant *Pseudomonas aeruginosa* treated with intraperitoneal aztreonam and peritoneal dialysis catheter removal. Despite broad-spectrum antibiotic therapy, the patient remained febrile. All microbiology laboratory tests were negative, so it was decided to stop antibiotic therapy for 48 hours and repeat cultures in order to avoid possible false negatives. In new blood cultures performed after suspension of antibiotic therapy, candidemia was observed, later typified as *Candida auris* species. After completing antifungal treatment (three weeks with intravenous amphotericin B 100 mg qd and two weeks of intravenous anidulafungin 100 mg qd), microbiological cultures remained negative and the patient made uneventful recovery.

**Conclusion:**

*Candida auris* invasive infection has been mainly described in patients with severe underlying comorbidities and immunocompromise. Multidrug-resistant clusters of *Candida auris* are increasingly emerging.

## 1. Introduction

Combination treatment with micafungin and voriconazole has demonstrated efficacy for these multidrug-resistant isolates, but in case of urinary tract or central nervous system infection, amphotericin B added to 5 flucytosine is preferred, always guided by sensitivity tests.

## 2. Case Report

A 57-year-old man with kidney failure secondary to autosomal dominant polycystic kidney disease (ADPKD) receiving peritoneal dialysis (PD) therapy was admitted to the reference transplantation hospital to undergo kidney transplantation. The patient's medication prior to transplantation included valsartan as an antihypertensive drug and intravenous iron. He received basiliximab, tacrolimus, and mycophenolate as immunosuppressive induction therapy. Twenty-four hours after the procedure, he complained of severe abdominal pain. The Doppler ultrasound confirmed arterial thrombosis, so urgent kidney transplantectomy was indicated and PD restarted. All immunosuppressive medications were immediately discontinued. During this admission, he developed *Pseudomonas aeruginosa* pneumonia that was treated with levofloxacin for 12 days and catheter-related *Enterococcus faecalis* bacteremia treated with linezolid for 21 days. After hospital discharge, he presented with herpes zoster at the lumbar dermatomes infection treated with valacyclovir for 7 days. Ten days later, he developed PD peritonitis and exit site infection with multidrug-resistant *Pseudomonas aeruginosa* (only susceptible to colistin and aztreonam). Since the patient remained asymptomatic, treatment was started on an outpatient basis with intraperitoneal aztreonam. However, poor clinical course was observed, with increasing acute phase reactants and persistent fever. Hospital admission was decided to complete the study and treatment.

On admission, antifungal treatment with fluconazole was started and stopped after 10 days due to liver toxicity. Colistin was also commenced due to the history of multidrug-resistant *Pseudomonas aeruginosa* peritonitis. In addition, empirical coverage with intravenous vancomycin and metronidazole was initiated, and a microbiological screen was requested including blood, urine, and peritoneal fluid culture.

Given the persistence of elevated acute phase reactants and fever, we decided to remove the PD catheter 7 days after starting antibiotic treatment, and the patient was transferred to hemodialysis through a right temporary femoral catheter.

Despite broad-spectrum antibiotic therapy, the patient remained febrile. All microbiology laboratory tests were negative. At this time, it was decided to stop antibiotic therapy for 48 hours and repeat cultures in order to mitigate possible false negatives. In new blood cultures performed after suspension of antibiotic therapy, candidemia was observed, later typified as *Candida auris* species. Given these results, right femoral catheter removal was decided, antifungal treatment combined with caspofungin and amphotericin B was initiated, and contact isolation of the patient was established. Subsequently, insertion of the right internal jugular temporary catheter was made to continue hemodialysis sessions. Caspofungin was suspended two days after initiation due to liver toxicity, replacing it with anidulafungin.

After initiation of antifungal therapy, progressive clinical improvement was observed, with persistent decrease in acute phase reactants and fever. Serum blood cultures were performed every 72 hours after the first week of treatment and remained negative in all determinations. Given these results, after one month of antifungal treatment, insertion of a right internal jugular permanent tunnelled hemodialysis catheter was performed.

After completing antifungal treatment (three weeks with intravenous amphotericin B 100 mg qd and two weeks of intravenous anidulafungin 100 mg qd) with negative microbiological cultures and given the clinical stability of the patient, he was discharged with subsequent outpatient follow-up.

## 3. Discussion

This is one of the few cases reported in patients on peritoneal dialysis and the first in our region. The newest *Candida* species called *Candida auris* was first reported in Japan in 2009 and named so as it was first described and isolated from the ear canal of a Japanese patient. It has been subsequently isolated from several body sites of patients in multiple countries on five continents [1]. It has been associated with nosocomial outbreaks in intensive care settings, and its transmission despite the implementation of enhanced infection prevention and control (IPC) measures is a particular concern.

The *Candida auris* genome is approximately 12.5 Mb [2]. Genome analysis suggests that there are between 6,500 and 8,500 protein-coding sequences, with a number of these genes coding for proteins characterized as virulence factors in other *Candida* species, such as biofilm formation [3]. In addition, multiple transporter genes and protein kinases, which may facilitate the acquisition of drug resistance, have been identified [2]. Colonization with *C. auris* has been detected at multiple body sites, including the nares, groin, axilla, and rectum [4,5], suggesting the need for systemic swabs/cultures and patient isolation after treatment and upon readmission to healthcare facilities.

*C. auris* has not only been associated with a variety of invasive fungal infections, including candidemia, sometimes related to central venous catheters, but also presents as pericarditis and respiratory tract and urinary tract infections [4,5]. Most of the cases have been described in intensive care facilities, usually in patients with severe underlying comorbidities including hematological malignancies and other immunocompromised states [6]. *Candida auris* infection treatment is a major concern due to the increasing emergence of multidrug-resistant clusters. Reduced susceptibility to triazole antifungals has been demonstrated in different regions, and there is also variable susceptibility of isolates to amphotericin B [7]. Consequently, echinocandins were initially recommended as empirical treatment. However, because of the widespread use of echinocandins, *C. auris* isolates with reduced susceptibility are appearing [8]. Combination treatment with micafungin and voriconazole has demonstrated efficacy for these multidrug-resistant isolates in vitro [9].

Mortality rates have varied significantly across geographic regions. As such, the overall attributable mortality rate is unclear. In the United Kingdom, no deaths were considered directly attributable to *C. auris* for 22 patients who received targeted antifungal treatment [6]. The number of deaths attributable to candidemia, as opposed to an underlying medical condition, may be difficult to quantify.

In conclusion, invasive *Candida auris* infection is rare, and one of the main determinants of prognosis is early treatment.
